# Progress of Cholera

**Published:** 1849-08

**Authors:** 


### PROGRESS OF CHOLERA.

Since the publication of our former number, the ravages of cholera in this country have been of an alarming character, and in meditating upon these fearful visitations, we feel that we cannot too strongly urge upon those who cultivate Medicine as a science, to institute the most rigid scrutiny into the habitudes of the Pestilence, so that it may no longer walk "in darkness" among the nations of the earth. The highest triumph of Medical Philosophy is in guarding the world from the visitations of Epidemic disease. In the midst of such a visitation, medical art is almost powerless; while approaching its acme, and until it begins to decline, therapeutics are almost futile. To the mind that narrowly observes facts, that gives each and every feature of a truth its exact bearing, and is not seduced from the path of philosophy by the fascinations of a theory, an epidemic is a fruitful source of instructive knowledge, and we beg of our readers, who may have such facilities, to treasure up facts and report them. In this way all may contribute to the advancement of professional knowledge, and to the safety of communities.

"Happy is he who knows the cause of things" was the sentiment of an eminent Latin writer, but in its application to the philosophy of medicine, it has a wider and broader signification than he gave it. In that application the community is blessed which has Physicians who know the cause of things and are able and willing to ward off a pestilence. We would not exchange the possession of such ability for all the ovations that ever awaited a conqueror, and the greenest laurel that ever encircled the brow of the victor.

From the bosom of nature springs the pestilence that desolates cities and country, and it is the province of mind to search into the mysterious agency and remove all doubts and speculations on the subject. We commend the whole subject to the attention of our readers, and assure them that, in the history of Epidemics, there is a field for exploration that cannot fail to enrich the minds of all who may carefully and prudently expend their energies in that direction.

At various periods of the world there have been many such pestilences as that of cholera, and, in our judgment, nothing can be plainer than the fact that nearly all of them were dependent upon a condition of things that were perfectly under the control of man, if he had set about the work properly. In the 14th and 15th centuries medical philosophy was scarcely advanced enough to make out the correct etiology for such epidemics as "black death," "sweating sickness," "dancing plague," &c., but even in the midst of the dearth of medical philosophy that then prevailed we gather very important and valuable facts that are highly useful in studying the phenomena of epidemic cholera.

In undertaking a study of the cause of epidemic disease, the following sources of knowledge must be thoroughly explored—*Physical geography,*—a science that has been most shamefully neglected—Meteorology, which, unfortunately, is yet in comparative infancy,—Physiology, Hygiene, and Pathology. An intimate acquaintance with each of these subjects of human knowledge is indispensably necessary in building up a system of Etiology, that may commend itself to the faith of mankind, and that may be useful to them. Groundless hypothesis, false systems, and erroneous data are never useful to the world; truth is the only consoler, and conservator, and is worth all other things.

In utter want of such knowledge, numerous speculators have recently appeared in the newspapers, delivering their inane dreams in the most perfect *ex cathedræ* style. A wild and untenable notion, that absence of atmospheric electricity is the cause of cholera, has been gravely advanced, and has attracted some attentien. We give this as a specimen of the grievous errors in existence. In some cases, when and where cholera was prevailing, electrical experiments failed, and the conclusion was made, not only that there was always a deficiency of atmospheric electricity during the prevalence of cholera, but that this deficiency was capable of producing cholera. The truth of the first does not necessarily prove the truth of the last proposition, but both were affirmed with equal zeal. Notorious facts should have settled both statements as wild and bootless guesses. Prof. Olmstead, of Yale College, has given the speculation its quietus by a series of philosophical facts, and closes his remarks with the following truth: "The disposition to ascribe the production of the cholera to variations in the electrical state of the atmosphere is not in accordance with the cautious spirit of the inductive philosophy."

One fact alone is sufficient to destroy the hypothesis: a deficiency of atmospheric electricity could not possibly be found in one or two spots of a large town, yet cholera often attacks a very small portion of a town, while all the rest remains heathy.

Cholera springs from a cause that is perfectly manageable, and we must seek a control of it, in an intimate knowledge of its nature. In a future number of the Journal, we intend to undertake an exploration of all the phenomena of malarial epidemics.

But we must take a hurried glance at the progress of cholera in the West. We shall confine our remarks to four western cities—Nashville, St. Louis, Cincinnati, and Louisville.

In Nashville, cholera commenced in January, after a flood tide of the Cumberland river, and was restricted almost entirely to the part of the city that had felt the influence of the water. There were but few cases in the winter; from the 1st of January to 1st of June, a period of six months, there were 210 deaths from cholera, while in June, the first summer month, the deaths from cholera amounted to 190—within 20 of the amount for the preceding six months.

In St. Louis, the ravages of cholera were almost beyond description. The meteorological phenomena, attendant upon its epidemic influence, were frequent rains and intense solar heat. These were frequently mentioned during the severest ravages of the disease. From the 2d of January to the 9th of July, 3,262 deaths from cholera are recorded, and from reliable information, we have no doubt that at least one-third of the deaths for several weeks were not registered. Burials were made in the Commons, and in private lots. Eight coffins were found in one pond.

Cincinnati has also suffered severely, and though we have no means of knowing the exact number of deaths in that city, we shall not err much in saying that the mortality there equalled that in St. Louis. The disease was confined at first to Texas ward and Mill creek, but it spread gradually to other parts. Heavy fogs at night enveloped the city, and malaria found in them a suitable vehicle for traveling. Neither St. Louis nor Cincinnati is well ventilated, and this violation of sanitary law, sufficiently accounts for much of the mortality. When malaria is rife, ventilation is a great protection from its noxious influence.

At a very early period in the spring, Louisville commenced her sanitary measures, and, thanks to the city authorities, and to the industrious and persevering efforts of the citizens, in the work of purification, she has passed the epidemic period almost unharmed. The Board of Health with abundant means of knowing, report 141 deaths by cholera; only about 100 of these were indigenous to Louisville, and not more than five or six persons, that were well known in the community, died. The rest were comparative strangers. These facts, occurring in a city of fifty thousand persons, show the value of well devised and well used sanitary regulations.

In the treatment of the disease here, we have found most success in the plan suggested by Dr. Ayre of England. In the early stages of the disease, a large dose of laudanum, say from 60 to 100 gtt., with a mustard bath for the feet was usually sufficient. Where collapse was rapidly approaching, we resorted to two-grain doses of calomel, with one drop of laudanum, given every five minutes, until biliary secretion was restored. This course saved many who were rapidly sinking under other forms of treatment. For the sick stomach, we found dry cupping, and sometimes scarification eminently useful, but occasionally administered one drop of creosote, in a tea spoonful of water, every hour. Ice and iced drinks were always useful, in every case we saw them used.

We have only space now for this meagre outline, but enough is said to direct the intelligent practitioner in his line of duty.

